# The Problem of Trust Without Intimacy: Education for Handling Expert Knowledge in a Neoliberal Marketplace

**DOI:** 10.1007/s11191-022-00329-z

**Published:** 2022-03-01

**Authors:** Dorothy V. Smith

**Affiliations:** School of Education, University of New England, Armidale, NSW Australia

## Abstract

Trust arises from confidence in a person or confidence in the practices of an institution. Theorists argue that institutional trust depends, to varying extents on intrapersonal trust, which is trust between people who know each other. Science rests its claim to expert knowledge on the practices of knowledge production engaged in by its institutions. Most people cannot check these practices themselves and effectively must trust the experts who explain and vouch for those practices of science, and thus, there is an element of intrapersonal trust needed if the laity is to have trust in science. Much of the sociology of science is concerned with democratic exchanges between scientists and other citizens, in which scientists are expected to show a commitment to open-mindedness and transparency, yet this may leave scientists and their knowledge vulnerable to contestation in terms that may undermine trust in their science. In this article, I draw on data generated in a study of Australian scientists to describe the ways in which trust was important in the work of these scientists and consider the consequences for a scientist who is prepared to admit to uncertainty. Drawing upon these data and from media accounts of the COVID-19 vaccination debate in Australia, I argue that science education for contemporary society must equip scientists and the laity for relationships that are more than narrowly cognitive. I argue for an education that makes explicit the ways in which the community of science interacts to produce and verify knowledge, and that equips students to recognise uncertainty and dissent as central to science and value expert knowledge. I suggest approaches that may achieve this goal.

## Introduction

Most people are more inclined to trust people they know than to trust remote expertise or an abstract system (Meyer et al., 2008). This tendency has been discussed in the sociology literature for decades (Fukuyama, 1995; Giddens, 1991a, 1994; Luhmann, 1993) and is an important problem because contemporary life in most societies requires that people have a degree of trust in science and technology even when they do not know scientists or are unfamiliar with the ways in which science works (Camporesi et al., 2017; M. Solomon, 2021).

The task of equipping the public to interact with science has formed an important strand in science and technology education, also for decades. In this article, I foreground the idea that considerations of trust in science education must acknowledge the ways in which people feel as well as how they think. Rationality is a valuable goal for education and it should be cultivated; however, “[t]he various studies of the ways in which adult members of the lay public engage with science suggest strongly that their interaction is rarely if ever narrowly cognitive” (Jenkins, 1997, p. 147). Faced with the need to trust scientific experts, the scientific laity find ways of dealing with the problems of the expert knowledge that may not include learning more science, learning different science or learning about science, and science education must acknowledge this. This idea has been repeatedly addressed in the science education literature (Feinstein & Waddington, 2020; Jenkins, 1997; Layton et al. 1993; J. Solomon, 2002, 2003) and I pick it up here acknowledging that in doing so I merely add another voice to this international debate. On the other hand, another voice in this debate may be of use.

In this article, I add to already published accounts of the professional lives of some contemporary scientists who work in Australia, a neoliberal marketised democracy. This analysis examines the ways in which preparedness to acknowledge uncertainty and limits to their knowledge impacted on their interactions within and outside science. The questions that I answer in this analysis are:

1. What do these scientists say about the relevance of trust to their work, including in work that brings them into contact with the public?
2. What are or might be the consequences for a scientist who is willing to admit uncertainty to another scientist or to a member of the public?
3. How might a school or university science education better support understanding of when and why trust in science is warranted?

The 2020 COVID-19 pandemic has made visible and intensified public engagement with Australian epidemiologists and other medical scientists. One consequence of the pandemic in Australia has been that the impact of market thinking on public trust in science has become easily discernible. For this reason, in addressing research question 3, I use the experience of the first eighteen or so months of the pandemic, in Australia.

In this article, I draw upon the thinking of Anthony Giddens, who, with Niklas Luhmann, is one of the most-cited thinkers about trust, and whose work particularly addresses trust between individuals and systems (Meyer et al., 2008). The formulation of trust without intimacy came to science education from Giddens, through Joan Solomon, who used the phrase to describe the need to trust the advice that we do not understand given by people we do not know or love (J. Solomon, 2002, p. 28). As Solomon describes it, there are two steps to the problem: science as an institution has developed abstract thinking to a level to which most people cannot follow; consequently, most people are forced into trusting scientists or the institutions of science without knowing the people or understanding the practices of the institutions, and people find this difficult to do.

For some two hundred and fifty years, since the time of the Enlightenment, science has pushed ahead with its usual emphasis on abstract thinking. … Gradually, the situation arose that those with knowledge became a smaller and smaller proportion of the population, but they became more and more difficult to follow. They were an intimidating force of experts, with knowledge so narrow and esoteric that most of the population did not even understand their explanations about scientific matters, and just had to trust them. Normally we give trust only to those in whom we have faith – shamans, priests – and those we love. Anthony Giddens identified this rather grim situation as ‘trust without intimacy’ and suggested that we could lose out if we cheapened trust in this way. (J. Solomon, 2002, p. 28)

The idea of trust without intimacy has particular immediacy given what we are learning about the post-truth era in the USA (Sismondo, 2017a, 2017b) and the complex interactions afforded internationally by social media (see, for example Bond et al., 2012). These are situations where trust appears to have as much to do with feelings as it does with rationality, and distrust of science appears to be tangled together with broad distrust of the institutions of government. Despite these difficulties, some contemporary scientists report working in complex ways to build trust through personal contact in quite unlikely situations, including with science deniers and sceptics (see, for example McIntyre, 2021).

Sociologists of science have been addressing matters of trust and governance between science and society, and scientists and their public for several decades (see, for example Funtowicz & Ravetz, 2001; Jasanoff, 2003, 2004; Nowotny, 2003; Nowotny et al. 2001). Implicit in these analyses and the recommendations they generated were accounts of societies, their democratic processes and the pre-occupations of their citizens. Here, I juxtapose Nowotny’s metaphor of the agora with that of the neoliberal marketplace, because it is becoming increasingly clear that some democratic societies, particularly at times of crisis, function more like a marketplace than an agora, transforming the meaning of trustworthiness in ways that may prove unproductive.

## Trust and Expertise

Giddens describes trust as “confidence in the reliability of a person or system, regarding a given set of outcomes or events, where that confidence expresses a faith in the probity or love of another, or in the correctness of abstract principles” (Giddens, 1991a, p. 34). Broadly speaking, the literature distinguishes between two forms of trust. The first of these is institutional trust, or as Giddens puts it, faceless trust; the second is interpersonal trust, or in Giddens’ terms, trust arising from facework (Fukuyama, 1995; Giddens, 1991a; Meyer et al., 2008). Interpersonal trust is “negotiated between individuals (a decision to trust someone or not) and [is] a learned personal trait” (Meyer et al., 2008, p. 178). Institutional trust is trust placed in an institution or an expert system, where expert systems are “systems of technical accomplishment or professional expertise that organise large areas of the material and social environments in which we live” (Giddens, 1991a, p. 27). Contemporary life in most societies, to varying extents, depends on trust in expert systems, and science and technology may be regarded as an expert system or a system of expert systems. Here, the idea of intimacy does not have to mean knowledge of a person: it can also mean sufficient knowledge of the practices of the expert systems. Institutions which broker scientific expertise, such as the National Health Service in the UK, have a key role in regulating and certifying expert systems (Camporesi et al., 2017).

Contacts with experts are “peculiarly consequential in modern societies” (Giddens, 1991a, p. 84) because such encounters are how the laity interact with the abstract principles of the expert system. In Giddens’ terms, these individual experts provide “access points” (Giddens, 1991a, p. 83) to the expert system and do the “facework” (Giddens, 1991a, p. 80) that contributes to “trust relations which are sustained by or expressed in social connections established in circumstances of copresence” (Giddens, 1991a, p. 80).

At access points the facework commitments which tie lay actors into trust relations ordinarily involve displays of manifest trustworthiness and integrity, coupled with an attitude of “business as usual” or unflappability. Although everyone is aware that the real repository of trust is in the abstract system, rather than the individuals who in specific contexts ‘represent’ it, access points carry a reminder that it is the flesh and blood people (who are potentially fallible) who are its operators (Giddens, 1991a, p. 80).

However, “[e]xperts can get things wrong, by misinterpreting or being ignorant of expertise they are presumed to possess” (Giddens, 1991a, p. 86). Giddens argues that if the public is to retain its trust in expert systems, then it is important that the operators or purveyors of abstract systems should maintain control over the access points to those systems and thus avoid the possibility that the public lose trust by becoming aware of the gaps and fragilities within the system. Logically, then, openness about uncertainty or the fragility of recently produced knowledge can undercut institutional trust.

There is no skill so carefully honed and no form of expert knowledge so comprehensive that elements of hazard or luck do not come into play. Experts ordinarily presume that lay individuals will feel more reassured if they are not able to observe how frequently these elements enter into expert performance. (Giddens, 1991a, pp. 86 - 87)

For Giddens, trust in expert systems is sustained by facework: a person must trust a physician in order to trust the medical system. By contrast, for Luhmann, people must trust the system before they can trust the system’s representative. Both theories of trust have been critiqued for being linear and failing to take into account the complex web of relationships that may influence a decision made by any individual to trust or mistrust a particular expert, technology or institution. Both men have also been critiqued as theorists whose ideas have not been sufficiently empirically tested (Meyer et al., 2008). However, both these theorists and others agree that trust is needed where there is inadequate information to rationally judge the consequences of a choice and that the decision to trust has an emotional or affective weight. For Giddens, trust “presumes a leap to commitment, a quality of ‘faith’ which is irreducible … not by any means always the result of consciously taken decisions … We can make the decision to trust … [b]ut the faith which trust implies also tends to resist such calculative decision-making” (Giddens, 1991b, p. 19). For Luhmann, trust always extrapolates from available evidence, but he recognises that rational accounts of trust can be constructed post hoc.

Although the one who trusts is never at a loss for reasons and is quite capable of giving an account of why he *(sic)* shows trust in this or that case, the point of such reasons is really to uphold his *(sic)* self-respect and justify him *(sic)* socially … At most, they are brought into account for the placing of trust, but not for trust itself. Trust remains a risky undertaking. (Meyer et al., 2008, p. 183)

## Expertise and Transparency in Science

Several types of answers have been given to the question of why science should be trusted. Some of these answers look to the people who work in science, either behind the scenes or more publicly, and argue that since these are people of good character, the explanations they produce must be true. Another type of answer looks to the utility of the products of science and argues that scientific theories must be true because they underpin technologies that work. These answers can be relatively easily refuted: the caloric theory of heat was successful in its time and produced several technologies but is no longer regarded as correct, and honest people may be honestly wrong (Oreskes, 2019).

A third type of answer to this question of why science should be trusted has focussed on the practices by which knowledge is produced in science. I use the term practices in the sense in which it is used by Evelyn Fox Keller, who described science as.

… the name we give to a set of practices and a body of knowledge delineated by a community - constrained although certainly not contained by the exigencies of logical proof and experimental verification. (Keller, 1986, p. 172)

In this sense, the set of practices that generate science includes the explicit and implicit aims and the values that drive them, the methodologies, techniques and rules and customs for checking and reporting, the method or methods by which scientific knowledge is produced and verified. Initial research into the practices of knowledge production in science laid emphasis on the philosophical problem of the basis for the truth claims of science (Popper, 1968) and later, building from Kuhn’s (1970) recognition that science was a socially embedded institution, the emphasis shifted to developing accounts of the ways in which scientists interacted within their scientific workplaces and with society more broadly (Jasanoff, 2004; Nowotny, 2003; Oreskes, 2019; Rose & Rose, 1969).

Thomas Kuhn’s (1970) theory of paradigm shift in science was also a paradigm shift in science studies as it shifted attention away from philosophical tests of truth to theories that build upon the social processes by which science knowledge is produced and verified. Considerable attention has been given to examining the processes by which scientists work together in and with institutions of science to produce, check, refine and extend knowledge that comes to be called science: for example Latour and Woolgar (1979) used methods of anthropology to study medical research in action and Latour (1987) argued from these and other studies that there was no singular scientific method (see also Oreskes, 2019). Arguably, these recent theories about science make it possible to believe that scientific truth is relative: Kuhn himself opened this possibility with his insistence that scientists were unlikely to agree across paradigms and that “there is no standard higher than the assent of the relevant community” (Kuhn, 1970, p. 94). For Kuhn, this community was that of science and it remains the case that scientific knowledge produced in the academy can be tested entirely within science.

However, contemporary scholarship in the sociology of science suggests that the practices of the expert systems of science are not as isolated from society as once thought. Many of these theories for scientific knowledge production have had a normative element, in that they recommend that scientists be more accountable for the consequences of their research and more transparent about its limitations. For example Jasanoff (2004) has argued that science and society co-produce each other; she has also argued for technologies of humility: “institutionalized habits of thought” (p.227) that acknowledge the limits of prediction and control in managing the processes and consequences of technoscience. Jasanoff argues that the survival of the public square depends crucially on the attitudes and actions of experts and calls for a fundamental reorientation of elite attitudes toward ordinary citizens. She is not writing here specifically about the scientific elite:

Civic learning in modern democracies demands a more robust understanding of what it takes for publics to know—an understanding we seem to have lost in the twenty-first century. It calls for reintegrating the techniques of fact making with procedures for engaging people on issues that are seen, once again, as mattering to their collective well-being. Those procedures will necessarily vary across political cultures, in conformity with long-held traditions of public fact making and deliberation, or what I have called “civic epistemologies”. The success of any such moves toward reviving the public square will depend, however, on a fundamental reorientation of elite attitudes toward ordinary citizens. (Jasanoff, 2021, p. S9)

Jasanoff is not the only theorist of the relationships between experts and their public to argue for a reorientation of elite attitudes towards citizens. The concept of mode-2 knowledge production was developed in the late twentieth century to express the idea that the production and use of scientific knowledge needed to be differently accountable to subject-matter experts, decision-makers and, crucially, citizens (Gibbons, 1994). Mode-2 knowledge is dispersed in its production, context-dependent, interdisciplinary and problem-focused, in contrast with mode-1 knowledge which is academic, investigator-driven and discipline-based (Poutanen & Kovalainen, 2010). Mode 2 knowledge is contextualised in the agora, a “problem-generating and problem-solving environment” (Nowotny, 2003, p. 156) populated by “arrays of competing ‘experts’ and the organisations and institutions through which they bring their knowledge and experience to bear on decisions taken,” (Nowotny, 2003, p. 156) and also by “variously jostling ‘publics’” (Nowotny, 2003, p. 156). The agora is described as a place “inhabited by a highly articulate, and never before so well-educated population” whose “[e]xperience of participating, at least in liberal western democracies, should also have taught [them] how to express their views and articulate their demands” (Nowotny, 2003, p. 151).

Knowledge that is produced in the agora acknowledges the local and the human. It challenges established habits of thought and is differently vulnerable to contestation from knowledge produced in the academy. Participation in the agora means that the scientist will necessarily be operating outside of the security of disciplinary boundaries and operating in this way increases his, her or their vulnerability (Nowotny, 2003). In the agora, there are many knowers and many knowledge pertinent to human life, and science is but one of these; here, scientists must answer questions they have not chosen and bring their knowledge to bear on issues that are not purely scientific or technical. They must address audiences that are never solely composed of fellow experts and increasingly must do so on the terms of their audiences:

Experts have to synthesise all available knowledge and of necessity transgress the boundaries of their discipline as well as the constraints of their own limits of knowledge. Frequently, they feel under pressure of having to act as if they knew the answers and the conditions under which the answers will unlock an unknown future. The right of experts to say in public “we do not know” has been won only recently. (Nowotny, 2003, p. 152).

However, the extent to which the metaphor of the agora should apply to contemporary liberal western democracies is moot. Many liberal Western democracies under neoliberal governance may also be described as marketplaces, in which knowledge is commodified (Krishna, 2014).

In the neoliberal marketplace, the value lies in commodification, and thus, what is valued is knowledge that can be traded independently of the knower. Here, publics are constituted as consumers, and the idea of an educated political actor is replaced with that of individualist economic anti-citizen (Carter, 2017, 2018; Krishna, 2014). Consumers in the neoliberal marketplace are also highly articulated, often well-educated as consumers, and have learned how to express their views and articulate their demands. However, the interaction between experts and these consumers is different from that of the agora. If science is being done for a neoliberal marketplace, and if scientists are positioning themselves competitively as knowledge purveyors, then they will face pressure to maintain their market share by giving the public what they demand.

In the marketplace, knowledge is judged on the basis of its utility to the market, which is a different thing from its utility to science. The market may choose to purchase doubt without regard to the authority of the source of that doubt. Oreskes and Conway’s (2010) study of rocket scientists who were employed to spread doubt of science describes an ironic inversion of scientific practice: scientists from outside a field were able to bolster confidence in the status quo by being certain about their doubt of findings produced by scientists within a field of science. Scientists who approach a neoliberal marketplace with humility and openness may find that their willingness to engage is turned against them and their expertise is diminished. Displays of manifest trustworthiness and integrity may be interpreted as the performance of a skilled salesperson; unflappability may be interpreted as a failure to care or a tendency to look to self-interest first.

## Science Education for the Agora and the Marketplace

Internationally, science education has seen a variety of movements over the decades, each of which has given different answers to the questions of the nature and purpose of a school science education (Fensham, 2021). Implicitly, each of these movements embeds an account of science and thus teaches students something about science while teaching the science. As an example, PSSC Physics, one of the “Alphabet” science programs developed in the 1950s and 1960s (Fensham, 2021), had a story to tell about the nature of light and matter, and disregarded areas of physics that did not fit its grand narrative. PSSC was developed at a time when the power of modern physics had been explosively demonstrated at Hiroshima and Nagasaki, and it was implemented in schools during the “Cold War” and the “space race”, yet the text gives no indication of these contexts and in PSSC physics is presented as an intellectual adventure (Author. 2007).

Subsequent movements have been more explicit about the connections between science and society, and what they hoped to achieve. The movements that perhaps most explicitly addressed the accounts of science produced by the social studies movements, discussed above, are Science-Technology-Society (STS) and its successors that approach teaching science through socio-scientific issues (SSI) (Levinson, 2012; Sadler, 2011a; Simonneaux, 2014a) and socially acute questions (SAQ) (Olivier et al., 2017; Simonneaux, 2014b). I make this claim broadly, acknowledging that these are broad umbrella movements, and there is “a penumbra of ambiguity” (Layton, 1994, p. 32) about the categories: the many slogan and labels include humanistic science (Aikenhead, 2003); science for citizenship (Cross Fensham, 2000; Osborne, 2000) and science for social justice (Bencze & Carter, 2011; Bencze et al., 2020). The movements are well-developed and of long standing, with decades of research and exemplary initiatives (Hadjichambis et al., 2020; Ratcliffe & Grace, 2003; Sadler, 2011b). What these movements and the innovations they generate have in common is the idea that science is socially embedded and explicit goals that school science should equip students to participate in a democracy, as distinct from a workforce.

SSI and SAQ curriculum initiatives take as their starting point a social issue and this issue generates the teaching of the socially relevant science. In many cases, the issue has environmental origins, perhaps inevitably given the significance of climate change to contemporary society, but also, perhaps as a reflection of societal concerns that coincided with the origins of the various STS-type initiatives (Fensham, 2021): for a contemporary example, a recent large international study examined the interaction between communities and species perceived as pests in New Zealand (France et al., 2017), France (Simonneaux & Simonneaux) and England (Christodoulou et al., 2021). However, societal issues are not restricted to the environment, and, for example knowledge has been produced and reported about teaching about risk in the context of surgical interventions for back pain (Levinson et al., 2011) and the use of argumentation in teaching about air quality (Simon & Amos, 2011). Amongst this variety, some curriculum initiatives explicitly equip students to handle the political vicissitudes of neoliberalism and the market (Bencze & Carter, 2011; Bencze et al., 2020; Carter et al., 2017).

Key slogans for international science education policy have been science literacy or scientific literacy, which Fensham described as “Competencies and understandings about science that persons can acquire through formal and informal education” (Fensham, 2005, p.541) and STEM, an acronym that usually means Science, Technology, Engineering and Mathematics (Hudson, 2021). Of these, science literacy or scientific literacy has been in use for several decades (Bybee, 2021) and has acquired a variety of meanings, including some that recommend that the purpose of learning science in schools should be that citizens are able to participate more fully or more meaningfully in society. Amongst others, Feinstein (2011) argued that science literacy should be useful to the citizen laity, and in a wide-ranging examination of the ways in which science education was implicated in relationships of power, Barton and Roth argued that science literacy should build social justice (Roth & Barton, 2004). By contrast, STEM Education is generally seen as “an opportunity to develop competencies in high-demand fields” (Hudson, 2021, p. 1) and has a function closely tied to the workforce, the economy and the market.

## The Context of This Study

In Australia, scientific research is funded by state and federal governments and by business. The Australian (federal) government funds research in universities by funding the universities and thus indirectly paying the salaries of academics who are expected to be research-active and directly through competitive research grants (Australian Academy of Science, 2021). University-based science research is also funded by business and some of this work is done on a commercial-in-confidence basis. The Australian Government also directly funds scientific research through organisations such as the Commonwealth Scientific and Industrial Research Organisation (CSIRO), an Australian Government agency responsible for scientific research and the Bureau of Meteorology (BoM), Australia’s national weather, climate and water agency. Australia is a federation of state and territories, and these states and territories also fund scientific research relevant to that state or territory through a variety of organisations and agencies: for example the Arthur Rylah Institute environmental research is an arm of the Victorian (state) Department of Environment, Land, Water and Planning (Arthur Rylah Institute for Ecological Research, 2021). Museums in each state are, on the whole, state funded.

Many state and federal scientific organisations also have an outreach and science popularisation role: as an example, the CSIRO has been active in school education for 40 years. In addition, many scientists have contact with the public as part of their role: for example radio stations make live crosses to BoM senior forecasters several times each day for weather updates; scientists doing research into water or soil may be invited to address interest groups, such as land care organisations and, of course, education is a key role for museums. Senior Australian scientists give policy advice to state and federal governments; some scientists also work closely with non-governmental organisations and lobby groups; some scientists have taken up an active role using social media platforms such as Twitter to disseminate their science and some also express personal political views on these platforms.

Despite this variety, Australian school science tends to give a highly academic account of science and STS, SSI and SAQ have not been the dominant slogans for Australian science education policy. Rather, science education policy in Australian schools has been driven by, successively, the “Alphabet” science programs, scientific literacy and STEM and the science curriculum in schools tends to be controlled by academic scientists employed in university science and engineering departments and experienced school teachers (Fensham, 2009). The STEM Education agenda in Australia has a strongly instrumental tone that is easily allied to a neoliberal agenda (Carter, 2017, 2018; Gough, 2015; D.V. Smith, 2021). STEM dominates Australian science education (Gough, 2021), although data show that this policy direction is not achieving the desired results, namely improved scores on international competitive testing (Gough, 2021) and a well-primed STEM pipeline that will ensure a ready supply of entrepreneurs (Carter, 2017) and workers for industry (Palmer et al., 2018).

The study reported here was underpinned by an argument that Australian science education could and should provide a more complete account of the work of contemporary Australian scientists, particularly those whose work brought them into contact with the public. Accordingly, an intended outcome of the study was to understand how scientists regard the members of the public with whom they interact and to articulate a framework for a socially responsible science curriculum that supported productive interactions between scientists and the broader community (Smith, 2011, 2021).

The possibility of human-induced climate change was the most significant science-related social issue being debated in Australia when these data were produced. The extent and sheer nastiness of the climate change debate became clear to us only after we had commenced the research and were seeking participants and arranging for the interviews. Then, we became aware that the acrimony of this debate impacted significantly on the daily lives and safety of some Australian scientists and consequently on their ability to do their work. Other contentious issues of the time included vaccination and water use; in Australia, vaccination is significantly more visibly contentious today than it was before COVID-19.

## The Study

The data reported here were produced in a qualitative study of 36 Australian scientists from six different states and territories. The scientists were all Australian in the sense that they had spent the bulk of their professional lives in Australia. All the scientists reported in this analysis had extensive contact with a variety of sectors of the public. They included those who worked for organisations such as the CSIRO, the Bureau of Meteorology, museums, at universities or at research institutes. Some worked across several such agencies: for example CSIRO scientists might undertake some university teaching and a university researcher also could be affiliated with an external research institute. Recruitment was both systematic and opportunistic: systematic in the sense that we approached the Human Relations departments of appropriate organisations and asked that an invitation to participate be distributed within the organisation, and opportunistic in that we contacted scientists with a significant public profile and asked them to recommend appropriate participants.

Most of the participating scientists were selected because we had reason to know or believe that their work brought them into contact with the public and they were still active researchers. There was a subgroup of scientists recruited for the study, nine in all, who were selected without regard to their public contacts, but rather because they taught a first-year science subject at an Australian university. We were interested in this group because of their potential influence in shaping the views of science held by Australian science teachers. Although we did not expect that this group of scientists would have significant involvement with the public, we followed essentially the same interview protocol with them as with the other scientists, and as a consequence we found that several of these scientists did, in fact, interact with a variety of groups or had done so in their career (Smith et al., 2015).

Data production for this study was by a one-on-one interview of between 90-min and 2-h duration. Diagrams and graphs were used as part of the interview (details of this approach are provided in Smith et al., 2013a, 2013b; D.V. Smith & Mulhall, 2015). The nature and extent of the scientist’s interactions with various public were established early in the interview by use of what we came to call a “Star Chart” (see Smith & Mulhall, 2015 for more details). Interviews were trialled and refined in consultation with some trial participants.

To protect participant anonymity and as ethically required by the researchers’ institutions, names have been withheld in reporting this research and replaced by pseudonyms. For the same reason, individual scientists have not been associated with a particular field of science, although a list of the fields in which the scientists worked has been provided.

The interview explored how the scientific work of each scientist involved interaction with the community and other groups; the skills required to communicate with those different groups, and how they developed those skills and how they saw the relationship between science and society. The interviews were transcribed and analysis was done by multiple readings for emergent themes, together with repeated listening to the recorded interviews, consistent with interpretive phenomenological analysis as described by Smith and Osborn (see, for example Smith & Osborn, 2008).

The scientists were not explicitly asked about trust nor about the ways in which the public reacted if they expressed uncertainty; however, several spoke about these things in the process of answering other questions, and these data have been identified through repeated readings of transcripts of the interviews. Thus, while in previously reported analyses the interview data were interrogated using a process of multiple readings for emergent themes, this analysis is different in that the transcripts were examined specifically to identify the comments made by participants that were pertinent to trust and uncertainty, again through repeated readings of the transcripts. Sample interview questions and an indication of the reasoning behind the interview structure may be found in Mulhall et al. (2017); D.V. Smith et al. (2015) and D.V. Smith et al. (2020); questions pertinent to this article are provided in an Appendix.

### The Participants

The scientists interviewed for this project nominated the following as their areas of scientific research, past or present: mineralogy; soil chemistry; toxicology; bushfire related research; taxonomy; entomology; forest management; surface science; zoology and water quality; behavioural and evolutionary biology; air quality; climate change and agriculture; water efficiency in farming; beam-line science; chemical techniques of drug delivery; gambling and games of chance; ecology, conservation and aquaculture; climate change; forensic science (crime scene research); forensic chemistry; chemistry and materials conservation; radio astronomy; chemical engineering; immunology and microbiology; earth systems science, climate change; environmental science, ecology, climate change; fresh-water biology and astronomy.

The scientists interacted with a wide range of groups. The largest number of different groups a scientist indicated interacting with was twelve and the smallest number was four: this smallest number was reported by an academic scientist who did contract research with industry. The groups with whom they interacted included: business, industry and finance groups; community groups and community educators; non-governmental organisations and special interest groups; citizen scientists; providers of essential services such as firefighting authorities, water, emergency services, health services; farmers, farming systems groups and farm consultants; the general public; government regulatory authorities and resource managers; Australian indigenous communities; land and site managers; non-scientist professionals, such as lawyers, engineers, architects; the media; policymakers, senior policy makers and government officials (international, federal, state, local); politicians and political parties; schools/school students/school teachers; scientists who work in different fields or disciplines to the scientist and social scientists. In addition, they interacted with a range of people within their organisation or allied with it. These included funding agencies/bodies; non-scientific colleagues who work in same organisation (e.g. marketing, management); non-scientists with expertise relevant to scientist’s area of interest; professional organisations involving scientists with similar broad interests; providers of digital technologies or other support for research; research organisations such as universities or the CSIRO; tertiary educators and university officials.

All the scientists were active in research, and although some no longer did research in science, all had been research scientists at some stage of their careers. All the scientists were communicators of science, in the sense that their work required them to explain, interpret or otherwise communicate an aspect or aspects of science to a non-scientist. None worked exclusively as a science communicator; however, several scientists provided opportunities for broad public interest in science, for example by giving public talks. The scientists worked with this wide range of public for a variety of reasons: some work on complex problems that require input from a variety of scientific and non-scientific sources; for others, direct contact to provide information to the public was part of their job. Some of these scientists engaged with the public in ways that were discretionary in addition to the contact that formed part of their work. Some of these scientists have a high profile across Australia, others work more privately. The length of time for which they had worked in science ranged from nine to fifty years (Smith, 2021).

### The Role of Trust and Uncertainty in the Work of These Scientists

The scientists in this study spoke of the importance of trust in the context of their work with other scientists and when working in cross-disciplinary teams or with fellow professionals. As one scientist told us “without trust and confidence, nothing really moves forward smoothly” (Smith, 2021, p. 183). Here, what was important was to be confident in shared goals or values and to trust each person’s expertise:

… we share a common ground in terms of our advocacy for good … policy, but, you know, there are … climate scientists and hydrologists and soil scientists and environmental economists, and all of those sorts of people … If we are working together … then I’m happy, as would they be, that we each contribute components of that document from our own expertise, and you trust the other one will know what they’re talking about. (Lara)

As has been reported elsewhere (Mulhall et al., 2017), the scientists placed a high value on openness and open-mindedness in interactions of this type. They spoke of scientific knowledge as provisional, in the sense that it had limits and also in the sense that empirical data might falsify a hypothesis. They saw a willingness to acknowledge the limits of their own knowledge as important for the processes of academic science (Smith et al., 2016). One scientist, Ivan, reported that he enjoyed not knowing: “I think (pause) part of what drives me is that sense of freedom, ah, that uncertainty is fine, um, and that it’s okay not to know”. At the same time, he reported pressure from within science to be certain and voiced satisfaction at eventually being proved right.

… one of my colleagues said to me, ‘Well what does it mean?’ when I’d just [collected the data]. And I said, ‘I don’t know.’ And he said, ‘Well you’re **fucking** (*sic*) useless!’ And I said, ‘Look, I’m collecting data, I’ll find out what it means at some stage … And so it took me 25, 28 years until I got to the stage where I could prove it to everyone that I was right – well, not that I was right, but that equations explained it. (Ivan, Ivan’s emphasis).

Trust and uncertainty played out differently in interactions with non-scientists, even when these non-scientists were experts in their own right. Several scientists in this study reported being aware that work they saw as tentative was likely to be interpreted as certain in these situations. This could be because the laity did not take into account the underpinning basis of the science. As Jared said of lawyers, “Effectively when the science gets to them, it’s a bunch of facts, and the basis of those facts is probably not particularly relevant”, while Perry described a desire on the part of the executives he dealt with to see things “in black and white”:

The concept of uncertainty is something, especially some of the executives, find hard to understand. They see black or white, and it’s not black or white”. (Perry)

The scientists expressed a view that it was important that the public trust science. Perry saw public trust in science as helping him, as a scientist, to make his point:

… if the perception … is that science can be trusted and then once it’s been substantiated, it’s much easier to make your point. … the reputation of scientists **can** … affect people’s opinion, and it does ... I think it does make a difference in a conversation. (Perry)

By contrast, Ivan spoke of an occasion on which his pre-existing relationship with a senior public official had smoothed his path to discussing science with that person:

I was able to establish confidence and trust directly in a one-to-one relationship with the [senior official] that in a way empowered me in a different way to my boss. (Ivan)

Ivan spoke at length about his work with Australian indigenous communities and did so in ways that indicated his openness to mystical and spiritual aspects of indigenous cultures, such as awareness of place and respect for the authority of elders and ancestors. He reported that his willingness to admit that he did not know had been of benefit in his interactions with such communities.

I came back from doing some measurements on this remote site, and the elder … said, ‘Well, what did it mean?’ And I said, ‘I have no idea.’ I said, ‘I got results that were totally unexpected, so … I know nothing, um, but the rocks have spoken to me’ (laugh). ‘They’ve given me their condition, it’s up to me to work out what it is, what their message is,’ and I said, ‘It will take me some time to work it out,’ and I said, ‘yeah, so I guess you could say I know nothing.’ And he said, ‘Oh the white fellas they come up from [the city], they reckon they know … everything, and they don’t. You reckon you know nothing – I reckon you know fuck (*sic*) plenty. Ah, you an honest man, you can come to my country any time, and we’ll look after you.’ (Ivan).

Tara felt that the public could be disappointed if she could not apply her findings to their context. She spoke of the trend becoming better with time as she became better known to the public:

I think that sometimes people are disappointed that you can’t automatically translate some science into a management strategy specific to [their context], and so you’re trying to ... you’re trying to generalise enough to be useful, but you also need to be truthful to your science training and some days are better than others. Sometimes it can be quite difficult, and so then the interaction becomes less effective because you can’t give them the specific answer they’re asking for because ... perhaps because of the specific measurements that you made that don’t really relate to their problem … you become better known with time and people learn that they can trust you and the things that you do say, and obviously again, you become more confident with experience, but you can have bad days because it becomes very difficult to be able to cover off all their needs within the scope of the work that you’re doing. (Tara)

Scientists did not always feel supported by the press and other media in accurately conveying scientific information. As Pierce said, “it’s a mixed situation with the public media”. Jack spoke at length about his frustration with the communications department of his organisation. Because he worked very closely with the public, Jack was very experienced in writing for a range of people who used his findings and he felt that the communications department would “misjudge the level of understanding that the audience would have”. Jack reported that one of the functions of the communications department was in fact to maintain oversight and control of the scientists’ interactions with the public.

… essentially we just have a whole department that’s designed to help us communicate with the outside world, and also approve what we say so everything that we say has to be ticked off by the communications department and you know your line manager or above, and also they’ll communicate back to us what other things are going on. (Jack)

The scientists were aware that not all their interactions were with people whose chief concern was community benefit. Jeff worked in an area in which risk is high and the consequences of product failure can be catastrophic. He interacted with people running businesses as well as with community groups and he reported that not all business-based interactions were alike:

You’ve got some guy that started a company and he’s trying to make a product that’s going to help the community, and he’s just so sincere and dedicated, whereas the next guy’s just completely focused on how many bucks he can make out of it, whether it works or not. (Jeff)

Kim expressed a concern that the dishonest behaviour of an individual scientist could damage public trust in science:

… there’s a particular person who will trot out formulas and numbers about level of risk … and so on. The formulas are all correct. The calculations are correct. But the numbers that are put in there are just totally unrealistic, and it’s just so dishonest, and this guy gets an enormous amount of press, and talks about all his years of … experience. Well, the last time he had [relevant] experience would have been thirty years ago, you know? He might have started ages ago, but he hasn’t had an ongoing connection with it. So yeah... Science gets so abused that you start to lose ... you know that scientific method has a value, but it’s certainly not absolute, and public don’t trust it. (Kim)

We asked the scientists “What do you make of people who challenge the findings of science?” Their answers ranged from “I think it’s human nature” (Max) to “it depends on what grounds they’re doing it on” (Jack) to “I think they’re playing a really important role in the scientific process” (Carl), while Prue gave an answer that distinguished between challenging the findings on the basis that “the science was not good science” and challenging the findings “for value reasons – not on the science [but] because … of a recommendation that comes out of science.”

Parker was one of several scientists we interviewed who had decades of experience in dealing with the public, and he had developed strong views about his role and the terms on which he should interact. He saw his role as presenting the science but not advocating for a particular point of view because advocacy on the part of scientists reduced public trust in science.

Trust is incredibly important … the greatest risk of anything is public outrage, and the only way you can avoid public outrage is being upfront, honest and gaining trust … one of the big issues we’ve currently got in science … is of almost a distrust of science, and that has been, to some degree, driven by political reasons, and certainly advocates of particular ways of life. And so there’s been a collapse of trust. (Parker)

Parker reported that he has faith in people, but also was aware that interactions needed to be carefully handled so as to avoid asking too much of a group. He told a story of being at a meeting at which scientific findings were, in his view, misrepresented by a woman he described as representing a “right wing think tank”:

… she was telling the irrigators and farmers … that in fact the [river] was improving ... And she was putting up all these graphs to demonstrate that this was true ... so one of the scientists there who was talking, and he said, look, I’ve told you that that graph is incorrect, but you keep on producing it. But what happened was, she told the audience everything they wanted to know … the guy that organised the meeting, said to me – why didn’t you say anything? And I said, well what’s the point? I said, it’s a completely closed meeting. Their minds are shut … and he said, well would you come and talk to them? And I said, no, not as an advocate. I’ll come and talk to them about what I know about my streams and my animals. And he said, oh that would be good.

I spoke to them and I talked about the [river], and what my animals told me about the [river] and its decline. I didn’t say that anyone was wrong or right, I just gave them the evidence, and I said – and now it’s up to you to make your decision … But they all came up to me and said that their views had changed, because I’d actually asked them to think about another way of looking at things, and [the organiser] said to me, he said, five years after that, he said they still talk about that night.

… Yeah, so I have a lot of faith in people ... misplaced occasionally, but not... I suspect the majority of people don’t want to be doing the wrong thing. (Parker)

## Discussion

The overarching goal of this research was to provide accounts of the interactions between contemporary Australian scientists and their public that could inform school education. The irony of this goal is that society changes constantly and continually and since science and society are co-produced, so does science; the Australia in which these data were produced is different from Australia today and so there is a need to continually refresh these accounts. The COVID-19 pandemic has meant that change has taken unforeseen directions and it is likely that the pace of change has been faster than otherwise; the work of epidemiologists and public health specialists has gained prominence; there have been fewer public meetings than before, and museums were closed for a long while. Thus, the answers I give below to the first two questions in this analysis should be seen as telling how things were at a particular time and place. By contrast, my answers to the third question are inevitably influenced by the experiences of the pandemic.

Despite these changes, there is durability in the finding that Australian scientists do, indeed, interact with the public in a wide variety of ways and it is worth looking further than the work of academic scientists, narrowly framed, to provide accounts of science for schools. Trust is important within science and between scientists and the laity and I argue below that the practices of the market risk undermining this trust.

### The Relevance of Trust to the Work of These Scientists

Trust is important within science because scientists need to be confident that their fellow scientists are reporting their findings fully and accurately. Trust is also important when scientists work with other experts in multidisciplinary teams, where members rely on each other for the necessary expertise. The scientists in this study reported cases in which other scientists were prepared to exaggerate the extent of their knowledge and this concerned them, at least in part, for the impact that it had on public trust in science. In Giddens’ (1991a) terms, these scientists understood that public trust in science depended on the transparent integrity of the expert system that is science; in Oreskes’ (2019) terms, they understood the need to be prepared to explain what they know and on what basis they know it.

Science also needs to be accountable to decision-makers and the public at large. To varying extents, scientists in this study engaged in approaches to knowledge production consistent with Gibbons, Nowotny and Scott’s vision of the agora: these scientists answer questions they have not chosen and bring their knowledge to bear on issues that are not purely scientific or technical. They addressed audiences that are never solely composed of fellow experts and frequently, although not always, did so on the terms of their audiences. As the comments made by Jared, Perry, Pierce and Parker show, these scientists had a fine understanding of the limitations and needs of their public, they understood the hazards of having their work taken up by members of the public who misunderstood the terms on which it had been created and they had developed strategies to avoid being misunderstood.

There is evidence in this study that scientists are willing to engage with the public in ways that allow the public to see that they care about science and care about the consequences of their work. These are glimpses of the person that contribute to building relationships of reciprocal respect (Smith, 2021), and which, in Giddens’ terms, does the facework needed to build trust in the scientist’s expertise and trust in science. Scientists held different views regarding the interplay between trust in science generated through the facework of scientists and trust in the scientist being a consequence of trust in science, with Perry arguing that public trust in the institution science supports his work and Jack, Tara and Ivan speaking of situations in which trust in the person allowed people to be willing to interact with science or persevere when science seemed not to be producing the desired outcomes.

### The Consequences of Admitting Uncertainty for These Scientists

The scientists in this study reported pressure to be certain from fellow scientists and from various sectors of the public. Such pressure may have a variety of motivations: for example in the case of Jared and Perry, it may be that scientific complexity is suppressed as a means of handling legal complexity. Ivan’s experience provided the most dramatic contrast, where a scientific colleague was impatient with him for refusing to speculate about the meaning of new data while an Australian indigenous elder regarded a similar statement as a reason to have confidence in Ivan. This is consistent with Carter’s (2017, 2018) point that there are more ways to interact then those of the market: Carter specifically identifies an indigenous Australian leader as calling for a broader vision.

Arguably, the ways in which people respond to a scientist’s assertion of uncertainty will depend upon their expectations and beliefs about science and on their purposes for interactions with scientists. It is possible that the pressure to be certain comes because of a perception that science can and should produce “unequivocal and absolutely precise answers” (Russell et al., 2010, p.38). The experiences that Jared, and Perry report, of needing to express their knowledge in “black and white” terms could also be read as pressure to refigure their knowledge on terms that are consistent with the needs of particular markets or particular consumers.

In the agora, where the intention is to include rather than exclude and to pursue an agenda of democratising expertise by pluralising knowledge, an admission of uncertainty may be read as openness. By contrast with the agora, the intent of a neoliberal marketplace is simply that of a market, namely a commercial exchange of knowledge as a product: here what is valued is ownership of and control over knowledge for long enough to turn a profit. In the marketplace, knowledge and uncertainty must be marketable and this constrains the forms they may take: the market is likely to value certainty over transparency even where the certainty is misleading. If this is the motivation, then a scientist who acknowledges uncertainty can risk problematising their own status as an expert, and problematising the status of their scientific knowledge. Oreskes and Conway’s (2010) study of science being used in the service of big tobacco and climate change denial provides a salutary example of the ways in which doubt may be commodified in response to market demand.

One consequence of the shift to the marketplace is that a consumer has the ultimate choice to buy or not buy knowledge that is on offer. In a marketplace, if a customer does not want a product, then they are free to shop around until they find something that is better suited to their needs or tastes. Responsibility for that choice lies with the consumer, as does responsibility for the consequences of that choice: the neoliberal state “installs apparatuses and knowledges through which people are reconfigured as productive economic entrepreneurs of their own lives” (Davies & Bansel, 2007, p. 248). In a marketplace, consumers may choose between competing theories based on which is most useful to their productive entrepreneurship; they may choose the one that makes the most sense to them rather than the one which is most trustworthy. There is evidence in this study that scientists were aware that not all members of the public are motivated by the public good: as Jeff reported, while all business owners need to make a profit if they are to stay in business, some also wanted to help the community while others were largely motivated by the thought of profit.

Logically, in a neoliberal marketplace, mistrust of expertise should be the default setting of the laity: where one person is buying and the other selling the buyer should, to protect themselves, treat all vendors with suspicion until their wares have been tested. In this situation, a trust may be gained through prior experience of the goods or a prior relationship with the vendor. Here, as Tara’s experience suggests, it may be trust in the person that persuades the public to persevere with the knowledge produced by a particular scientist even though their immediate needs do not appear to be met. Here, also, Parker’s decision not to directly attack the views of a speaker who had already won the trust of the audience but rather to approach the audience on another occasion demonstrates a fine understanding of his public.

### Science Education that Better Supports Understanding of When and Why Trust in Science Is Warranted

This discussion of ways in which Australian science education might educate for rational and productive interactions between scientists and the laity is framed by Jenkins’ (1997) point that interactions between the public and science are rarely narrowly cognitive, and the work of science educators who recognise that learning more science may not be the best approach to creating a science-literate society (Feinstein & Waddington, 2020; Jenkins, 1997; J. Solomon, 2002, 2003). Rather, it may be necessary to choose to teach different things about science and the work of scientists than we do at present. To be clear, I am not recommending that students should learn no science content knowledge in schools. Rather I am advocating a particular take on approaches that already have been explored in the movements to teach science through socio-scientific issues (Hodson, 2020; Sadler & Zeidler, 2004; Simonneaux, 2014b).

Oreskes (2019) argues that in the end science and scientists should be trusted for two reasons. The first reason is because of science’s sustained engagement with the world. Just as a plumber should be trusted to do plumbing and a dentist to care for our teeth, so “to the extent that we should trust anyone to tell us about the world, we should trust the scientists” (p. 56). The second reason is “the social character of science and the role it plays in vetting claims” (p. 57). The process of vetting involves both dissent and consensus: “It is through the give and take of ideas – the challenging, the questioning, the adjusting and amending – that scientists integrate their colleagues’ work, offer up criticisms and contribute to the growth of warranted knowledge” (p. 51). Both these reasons are logical, true and valid. The difficulty is that although people should trust science for these reasons, it is becoming increasingly clear that, despite these reasons being true, science is not trusted as it might be. Rather, people trust for a variety of reasons and show a preference for advice given by people with whom they have a personal connection (Bond et al., 2012). Emotions, feelings and opinions, especially if they match what people already believe, can matter more than facts, and these choices are facilitated by a loss in respect for and trust in experts (Sismondo, 2017a). Parker may have been correct in that most people were well-intentioned when it comes to choosing their actions; however, it can be difficult for people to judge what is the right thing and an incorrect explanation that makes sense is likely to be preferred over a correct one that does not. Science may be challenged for rational reasons but also, as Prue pointed out, because people disagree with the underpinning assumptions and values.

Education could and should teach students about the need to trust knowledge produced and transmitted by others. School already teaches this implicitly because students depend on their teachers to prepare the learning environment. Even in science classrooms where students develop an understanding of investigation and science methods through metacognitive approaches, the students learn a good deal in addition to what they discover for themselves.

Classroom science does not rediscover and justify our scientific knowledge anew for each group of students. Most of science content - which is to say, most of science education - is taught through trust: trust of teachers, of textbooks, and of other pedagogical tools such as educational videos and other kinds of science literature. (M. Solomon, 2021, p. S37)

It is entirely possible to examine who else should be trusted about science and for what reason. The process would start by uncovering the wide range of formal and informal sources of information about science and examining the basis on which people adopt their positions and make their decisions, including an acknowledgement that while it is important that important matters should be decided rationally, it is entirely possible that this will not happen (France et al., 2017; Levinson et al., 2011). It is important that we provide ways to interact with science that are accessible to humans, who can struggle to trust people they do not know especially on matters they do not understand.

There is space for education to explicitly acknowledge the reasons why people might disagree with the findings and recommendations of science and the ways in which communities can work with scientific experts to understand and take social and political action (Bencze & Carter, 2011; Bencze et al., 2020; Roth & Lee, 2002). Solomon once argued in the context of England that “… about forming groups, whether it is for playing music, riding motor bikes, or pursuing political action, [young people] do not need to be taught. They know” (J. Solomon, 2002, p. 29). However, I suggest that while the laity does know about forming groups “of like-minded people” to bolster “identities through social means and amass enough support to gain knowledge and call on the government to act upon it” (p. 29), these groups will not necessarily engage productively with science. On the other hand, productive engagement between science and society has been shown to generate a communally situated science literacy of praxis in the USA (Roth & Barton, 2004; Roth & Lee, 2002) and teaching about such engagement using local examples potentially has value in Australia also.

Science education should make explicit the ways in which the community of science interacts to produce and verify knowledge. This would mean that students are given accurate accounts of contemporary science rather than the idealised accounts of individual heroes that have tended to be offered in the past (Allchin, 2003; Milne, 1998). This recommendation has a particular priority for teachers and curriculum developers with a commitment to fostering democratic exchanges that are broader than those of a market. The market is a dehumanised space and it is important to the neoliberal marketplace that knowledge is seen as disembodied because disembodied knowledge may more readily be commodified (Rose & Rose, 2014), and in a globalised marketplace, it is equally important that knowledge be minimally dependent upon geographical and social context. Teaching that science is done by people and in social and historical contexts is an important counter to the idea of disembodied knowledge (Smith et al., 2020).

Uncertainty is a normal part of science and so it is necessary that a scientist be allowed to be uncertain and to express this uncertainty. It is important that the increased openness of science does not compromise this ability. It is important that a scientist is able to say “I do not know” about the variety of meanings that scientific knowledge has outside the academy without compromising his or her claim to expert knowledge about the specific meanings that science knowledge can have in specific contexts. There are many meanings that might be taken from the phrase “I do not know”: for example it may mean “I do not yet know, but I expect I will someday”, as with Ivan’s exchange with his colleague; it may mean “I do not know because that information lies outside of my sphere of expertise” or it may mean “As a scientist I do not know because your question requires the exercise of knowledge and values outside of science”. None of these answers should problematise the scientist’s expertise nor invalidate what they do know; however, for this nuanced interchange to be possible, it must be understood and respected. Supporting students to develop the ability to understand these nuances should be a goal of education in schools and universities.

Science education should normalise scientific disagreement and students should learn about the processes and practices by which normal contemporary science proceeds, locally and internationally. Dissent is central to science: “scientific disagreement should not be interpreted as an indication that something has gone wrong, but, rather, that science is proceeding as usual, exploring various options in areas of uncertainty” (M. Solomon, 2021, p.S38). Solomon is writing about the education of scientists, who will learn these things alongside the knowledge and practices necessary for their future expertise. I suggest here that this approach is equally valid for the future citizen laity and arguably more important for them than the limited science content knowledge of a marginal insider (Feinstein, 2011), especially if that limited knowledge tempts the marginal insider to disregard relevant expertise.

### Trust and Choice in Australia During COVID-19

The COVID-19 vaccine debate in Australia in early 2021 provided vivid illustrations of the ways in which trust in experts interacts with choice in a neoliberal marketplace. I discuss one aspect of this debate here. I stress that this debate is not by any means completed, and consequently, this constitutes a partial and anecdotal account; nevertheless, these are the lived conditions under which I considered what might constitute appropriate science education for Australia in the future.

The Australian public response to vaccination was not a simple dichotomy of pro-vaccine or anti-vaccine; rather, it was a matter of which vaccine. In Australia, two types of COVID-19 vaccines were being used (Department of Health, 2021). One type was a viral vector vaccine, developed with a technology that has been well established for years, produced by AstraZeneca and manufactured in Australia. The other type was an mRNA vaccine, a relatively novel technology. Public preference across Australia was and is for the mRNA vaccine and this proved to be a significant problem for the Australian government because the mRNA vaccine was in short supply (Cormack et al. 2021). The demand for access to the mRNA vaccine was strong despite clear advice from experts that the AstraZeneca vaccine was effective against the currently dominant strain of COVID-19 and that having a vaccine—any vaccine—was better than being unvaccinated (Koehn, 2021). Heavy reporting of the side effects of the AstraZeneca vaccine may have been a factor in generating mistrust in the technology that could not be mitigated by accurate reporting of the relative risks (Gitau, 2021) and reassurance from medical experts was greeted with suspicion as to their motives.

What was notable about the discussion was the extent to which technical scientific information was being accessed by the press and by the public, including on social media (see, for example Doherty, 2021). What was also notable was the extent to which explanations about science written by reporters—as distinct from scientists and science communicators—were included in established media sources: this has resonances with Jack’s description of media specialists as misjudging the understanding of their audience. There were instances in which the explanations were clearly damaging to trust. To give one example, an opinion piece ostensibly discussing policy in a well-respected newspaper described scientific modelling developed by the Burnet Institute, one of Australia’s pre-eminent medical and public health research organisations, as “ultimately, sophisticated guesses – the modern version of soothsayers reading the future in chicken entrails” (Mannix, 2021, p. 4).

When the data produced in this study is examined through the lens of a COVID-19 pandemic, it becomes apparent that science education should, amongst other considerations, teach humility. Jasanoff argues for technologies of humility by which scientists in their institutions remind themselves of the limits of their understanding; I argue here for a corresponding humility of the laity through which we might come to understand the limits of an individualised intelligence. It is impossible for every citizen, as an individual, to handle every socio-scientific issue that they will encounter in their adult lives: this is true whether that individual is a scientist or not, because experts are not experts outside their particular area of expertise. Science education should teach this. If, instead, we transmit to students in our schools and universities the expectation that each person should make up their minds on socio-scientific issues without assistance, then we risk sending a message that the view of an inexpert individual carries equivalent weight to that of an expert. This is a dangerous message that diminishes the authority of science. School science education should acknowledge the special expertise of science and acknowledge that science is one of many areas of expertise. It should provide clarity about the power and limits of science in solving socio-scientific problems. Rather than encouraging students to make up their own minds on the validity of the science that applies to socio-scientific decisions, I suggest that it makes more sense to teach them how to judge who might be considered trustworthy and in possession of the appropriate expertise to advise on these matters.

## Conclusion

The data produced in this study and reported here show trust operating between Australian scientists and other citizens in a variety of ways. Scientists spoke of the need to trust the expertise of others when working in multidisciplinary teams yet also reported instances in which scientists exceeded the limits of their expertise; scientists spoke of public trust in science as important to their ability to interact with the public, and they also spoke in ways that suggested that public trust in the scientist as a person helped to build trust in science: this is consistent with Giddens’ description of the role of an individual expert in building trust in the expert system.

Public trust in science requires trust in the expert system that is science. That trust is mediated by the individual experts who provide points of access to science and who provide a human face for science. The work of these scientists reduced the need for the public to place trust in people they did not know and may not have met.

The experiences of these scientists raise questions about the extent to which knowledge produced for the agora is compatible with the neoliberal marketplace. Neoliberalism governs into existence a view of the public as consumers that is adverse to a vision of the agora as a place for democratic problem-solving. Neoliberalism now shapes public expectations of democratic participation and the terms on which knowledge is created, assessed and challenged. It demands that the first impulse of the laity should be mistrust rather than trust. Contemporary scientists who work with the public must handle interactions that are shaped both by the expectation of democratic exchanges between equals and also by the expectations of an impersonal marketised exchange.

School science should teach future citizens to critically evaluate who might be considered trustworthy, who possesses expertise pertinent to the issue at hand, and to recognise that sometimes a disagreement is about values rather than about science. They should also understand that dissent and uncertainty are fundamental to science and that scientific disputes are decided rationally, on the basis of logical proof and experimental verification. However, they should also be taught to critically assess debates about science within society, to recognise when science is being tested in ways that are emotional and to choose if and how they participate in such debates.

If our intention is to educate future citizens to participate in the practices of democracy, then school and post-school science education must acknowledge the possibility of interchanges between scientists and the laity broader than those of the market. For these exchanges, it is necessary that the laity learn the importance of humility in the face of expertise, a necessary counterpoint to the humility required of the scientist that recognises the limitations of their own expertise. It is important for the future disciplinary well-being of science and for the sustainability of life on this planet that science education equips scientists and non-scientist citizens to understand the limitations of the interactions of the market and to go beyond these.

## Data Availability

NA.

## Examples of Interview Questions

Early in the interview the scientist has annotated a diagram to show the various groups in the community with whom they interact in the course of their work or have interacted during their career.

**Interviewer: Let me try to summarise what we’ve been talking about. These are the groups you have identified, and I’ve written them on this page with science groups on the left and the other groups on the right.**

### Has the conversation with the people on the RHS of this chart given you different ways of looking at your science?

I’d like us to use this diagram to explore the ways that you see the groups on each side communicating with each other.

## Interviewer adds scientists in general to the list on LHS and says

In order to help with this I’d like to add … to the list on the left hand side of this diagram.

A. *What competencies, attitudes, skills do the people on the left need to enable a productive conversation with the people on the right?*
B. *What competencies, attitudes, skills do the people on the right need to enable a productive conversation with the people on the left?*

## Notes for the interviewer:

### Probe:

- Do they think people understand the work that scientists do? What makes them think that? If deficit model explore how they might come to understand better.
- Does doing science require a special point of view?
- View of people who challenge the recommendations of science?
- Has the conversation with the people on the RHS altered the way the people on the LHS see science?
- Do you think you have a role in helping people understand science (as distinct from your own work)?

## Code Availability

NA.

## Code Availability

NA.
